# Inductively coupled plasma mass spectrometry method for plasma and intracellular antimony quantification applied to pharmacokinetics of meglumine antimoniate

**DOI:** 10.4155/bio-2021-0013

**Published:** 2021-04-08

**Authors:** Diana J Garay-Baquero, David E Rebellón-Sánchez, Miguel D Prieto, Lina Giraldo-Parra, Adriana Navas, Sheryl Atkinson, Stuart McDougall, Maria Adelaida Gómez

**Affiliations:** 1 Centro Internacional de Entrenamiento e Investigaciones Médicas, CIDEIM, Cali, Valle del Cauca, Colombia; 2 Clinical & Experimental Sciences, Faculty of Medicine, University of Southampton, General Hospital Southampton, SO16 6YD, Southampton, UK; 3 Universidad Icesi, Cali, Valle del Cauca, Colombia; 4 Arcinova, Alnwick, Northumberland NE66 2DH, UK

**Keywords:** antimony, ICP–MS, leishmaniasis, meglumine antimoniate, PBMCs, plasma, TMAH, validated method

## Abstract

**Background:**

A high-throughput method using inductively coupled plasma mass spectrometry (ICP–MS) was developed and validated for the quantitative analysis of antimony in human plasma and peripheral blood mononuclear cells from patients with cutaneous leishmaniasis undergoing treatment with meglumine antimoniate.

**Materials & methods:**

Antimony was digested in clinical samples with 1% tetramethylammonium hydroxide/1% EDTA and indium was used as internal standard. Accuracy, precision and stability were evaluated.

**Conclusion:**

Taking the lower limit of quantitation to be the lowest validation concentration with precision and accuracy within 20%, the current assay was successfully validated from 25 to 10000 ng/ml for antimony in human plasma and peripheral blood mononuclear cells. This protocol will serve as a baseline for future analytical designs, aiming to provide a reference method to allow inter-study comparisons.

**Lay abstract:**

Cutaneous leishmaniasis is a disease caused by single-cell parasites in the genus *Leishmania* which results in painful skin ulcers and is spread by insect bites. Drugs containing antimony are the mainstay therapy for cutaneous leishmaniasis, but if and how the amount of these compounds in the cells can affect the success of the treatment, remains unknown. Validated methods to reliably measure these amounts in human cells are limited. Here we have developed a validated method that allows quantifying antimony in human plasma and peripheral blood cells from patients undergoing antileishmanial treatment. This protocol will serve as a baseline for future studies aiming to understand how antimonials work to treat leishmaniasis infections and how this therapy can be improved.

Leishmaniasis is a group of diseases caused by protozoan parasites of the genus *Leishmania,* which can cause cutaneous, mucosal or visceral manifestations depending on the infecting species and the host immune status [1,2]. According to the most recent report from the WHO, leishmaniasis is endemic across all continents. Despite an elevated proportion of suspected underreporting, 700000 to over 1200000 new cases are estimated to occur annually [2,3].

*Leishmania* is an intracellular parasite that invades and replicates predominantly within phagocytic cells (primarily macrophages), providing a ‘protected’ environment against host defenses and a barrier to drug exposure. Antimonial drugs are the mainstay treatment for cutaneous leishmaniasis (CL) of which systemically administered pentavalent antimonials (Sb^V^) are widely used in the Americas and as intradermal injections in the Old World [4]. Sodium stibogluconate (Pentostam^®^) and meglumine antimoniate (Glucantime^®^) are the synthetic compounds used for pentavalent antimonial therapy. Their efficacy is highly variable, according to the infecting species, parasite subpopulations, host responses and therapeutic regimens [5–7]. Antileishmanial drugs need to be distributed to the parasite-targeted tissues (primarily skin, liver, spleen and bone marrow depending on the infecting species), internalized into host cells and distributed to the phagolysosomal compartment where the parasite resides [8]. Although antimonials have been used over decades, the pharmacokinetics (PK) and pharmacodynamics of these drugs are not completely understood [9,10]. Few studies have described antimonial PK in the context of human leishmaniasis [11,12] and the plasma and intracellular PK parameters involved in the therapeutic response remain unknown.

Atomic absorption spectrometry is the most common method used to quantitatively determine antimony levels in biological specimens such as blood and urine from patients undergoing antimonial therapy in PK studies [11,13]. Although, a validated analytical method for quantification of arsenic and antimony in liposomes was previously developed using inductively coupled plasma-optical emission spectrometry [14], there is not a validated method available for intracellular antimony determination. Inductively coupled plasma mass spectrometry (ICP–MS) is an ultrasensitive and selective method that allows quantification levels in the order of parts per trillion, with a wide dynamic range of nine orders of magnitude and high throughput [15]. Interferences are relatively low and matrix effects can be minimized by using internal standard controls [16,17]. ICP–MS has been widely used in PK studies in humans of metal-based formulations such as arsenic, gadolinium and some antineoplastic drugs [18–22]. Its convenience and reduced time-consumption make this technique a powerful tool for quantitative determination of antimony in clinical samples at intracellular levels.

In this study, we present a scientifically validated analytical protocol for determination of total antimony in human plasma and peripheral blood mononuclear cells (PBMCs) from patients with CL undergoing treatment with meglumine antimoniate. The proposed structure for the meglumine antimoniate in aqueous solutions is presented in Figure 1 [23].

## Material & methods

### Reagents

ICP grade antimony (Batches 884136D and BCBP51619V) and indium (Batch 833920k) were obtained from Alfa Aesar (Heysham, UK) and Sigma Aldrich (Buchs, Switzerland). Electronic grade tetramethylammonium hydroxide (TMAH) was purchased from Alfa Aesar and EDTA from (Sigma). Human plasma for validation studies was obtained from Biochemed (Winchester, VA, USA). High purity (>99.999%) argon gas (BOC Gases, UK) was used for ICP–MS analysis. ASTM Type I ultra-pure water (>18 MΩ) was provided in-house by an Elga Purelab-Ultra system (Veolia Water Technologies, UK).

### Clinical samples & study design

Plasma samples and PBMCs were obtained from EDTA anticoagulated blood from patients with CL (n = 5). All CL patients had parasitological confirmation of infection and were recruited at Centro Internacional de Entrenamiento e Investigaciones Médicas (CIDEIM) outpatient clinics in Cali, Colombia. CL patient samples were collected from a cohort study that evaluates the relationship between antileishmanial PK, immune response signatures and clinical responses (CIDEIM IRB Study code CIEIH-1258). The study was reviewed and monitored by CIDEIM ethics committee in accordance with national (resolution 8430, República de Colombia, Ministry of Health, 1993) and international (Declaration of Helsinki and amendments, World Medical Association, Fortaleza, Brasil, October 2013) guidelines.

Participants were treated with intramuscular meglumine antimoniate (Glucantime [81 mg Sb/ml]; (Sanofi-Aventis, France) during 20 days with a dose of 20 mg/kg up to a maximum dose of 1620 mg of Sb(V), equivalent to a 20 ml injection. The median adherence to the indicated regime was 100% (95–100%). Peripheral blood samples were collected during the last day of treatment at the following time-points: before dose and at 0.5, 1, 1.5, 2, 3, 5, 8, 12 and 24 h after drug administration. In total, samples from 5 CL patients were included in this study (Table 1). All patients cured after treatment, and mild adverse drug reactions were reported by most study participants as shown in Table 1.

### Plasma procurement & PBMC isolation

Venous blood was collected in EDTA vacutainer tubes and plasma was prepared by centrifuging 10 ml of blood at 1300 × g for 10 min at room temperature. An aliquot of 3 ml of plasma was transferred to a fresh tube and centrifuged again under the same conditions. The plasma was transferred to a new tube and stored at -80°C. Remaining plasma and blood cells were gently mixed with phosphate-buffered saline (PBS) to complete 20 ml final volume. Blood/PBS mix was carefully layered on top of 10 ml Ficoll-Hypaque 1077 gradient (Sigma) in a 50 ml centrifuge tube and centrifuged at 400×g for 35 min at room temperature (RT = 19–23°C). The plasma layer was carefully removed, and the mononuclear cells layer was transferred to a separate centrifuge tube. Cells were washed with at least three volumes of PBS. Cells were subsequently centrifuged at 400×g for 15 min at RT. Then, the supernatant was discarded and the pellet was resuspended in 200 μl of 25% TMAH and stored at approximately -80°C until use. This PBMC preparation is referred as PBMC matrix for method development.

### ICP–MS analysis

The analyses were performed in a dedicated ACDP (UK Advisory Committee on Dangerous Pathogens) Class II bio-facility and all sample processing and digestion was performed inside Class I HEPA filtered biosafety cabinets to minimize the risks of contamination. All elemental reference standard preparations (Sb and In) were performed in a separate and dedicated laboratory to further eliminate the potential for sample contamination.

### Instrumentation & system suitability

Antimony and indium (internal standard), each have two stable isotopes: ^121^Sb and ^123^Sb, and ^113^In and ^115^In, respectively. Of these, ^121^Sb and ^115^In are most abundant and, therefore, were used to quantify antimony and indium, as internal standard, by ICP–MS.

All measurements were performed using an Agilent 7700x series ICP–MS, and used to quantify antimony (mass 121) relative to the indium internal standard (mass 115), following infusion of the sample into the ICP–MS via an integrated sample introduction system (ISIS) and micromist nebulizer. The operating system was the ICP MassHunter, version B.01.03 (Agilent, Stockport, Ches, UK). Operating parameters are listed in Table 2. The regression and quantitation procedures were performed using Watson LIMS version 7.5 SP1 (Thermo Fisher Scientific, MA, USA).

Prior to initiating any run on the system, an ICP–MS tune test was performed by analyzing a calibration mix containing cerium, cobalt, lithium, magnesium, thallium and yttrium (10 p.p.m., Agilent) in Tune mode. The raw counts should exceed 1000 units for each element and the variability (%RSD) should be under 10% for the system to be classified as suitable. Additionally, a system suitability test (SST) was executed by running two antimony dilutions; the low SST solution was 0.5 ng/ml and the high SST solution was 200 ng/ml. These dilutions were prepared using the indium internal standard solution (InISS) as matrix. InISS was a dilution at 2 ng/ml of Indium in 1% TMAH/1% EDTA. The blank was a dilution of 2% human plasma matrix in 1% TMAH/1% EDTA. The raw antimony count of the blank must be ≤25% of the low SST raw count.

### Preparation of calibration & quality control standards

Calibration standards were prepared by mixing an aliquot of the commercial antimony stock solution with purified water to yield eight calibration standards 2.5, 4, 25, 50, 250, 500, 725, 1000 μg/ml. Similarly, a set of five quality control (QC) standards were prepared separately with purified water to produce 2.5, 5, 50, 750, 10000 μg/ml concentrations.

### Preparation of calibration samples

Calibration stock standards were mixed with filtered control human plasma in a rotary mixer for at least 30 min at 30 r.p.m. to yield nominal antimony concentrations of 25, 50, 250, 500, 2500, 5000, 7500 and 10000 ng/ml. Calibration samples were either used fresh or stored frozen at either -20°C or -80 ±10°C for no longer than 56 days. Calibration samples used to determine stability were freshly prepared.

### Preparation of validation samples

Plasma validation samples were prepared by mixing QC standard solutions with human plasma in a rotary mixer for at least 30 min at 30 r.p.m. to give nominal antimony concentrations of 25 (quality control sample lower limit of quantification [QCLLOQ]), 50 (quality control sample low concentration [QCL]), 500 (quality control sample mid-concentration [QCM]), 7500 (quality control sample high concentration [QCH]) and 100000 ng/ml (dilution quality control sample [DQC]). Validation samples were stored frozen at either -20 or -80 ±10°C for no longer than 56 days.

PBMC validation samples were prepared by mixing an aliquot (100 μl) of the plasma validation samples QCLLOQ, QCL, QCM and QCH with and an aliquot of control PBMC matrix (100 μl) at the time of sample digestion.

### Carry-over assessment

Carry-over for the assay was detected during the assay development and was assessed during the assay validation. To circumvent any influence of carry-over affecting the clinical analysis, a blank sample was analyzed between each study sample. This procedure was tested in validation and the carry-over when using this approach was 14.7% (acceptance limit was 25%). For carry-over testing we analysed the following series of validation samples DQC (10000 ng/ml), blank and QCLLOQ-1 (25 ng/ml) and QCLLOQ-2 (25 ng/ml). We calculate impact DQC has on the difference between QCLLOQ-1 and QCLLOQ-2. In support of the clinical study we assess carry-over at the beginning and end of every batch (and reject if carry-over failed).

### Antimony digest preparation

Analytical samples were thawed at room temperature and vortex mixed. For plasma analysis, an aliquot of each plasma sample (100 μl) was mixed with 4.90 ml of InISS in a rotary mixer for 30 min at room temperature. For PBMC analysis, an aliquot of each PBMC sample (100 μl PBMC matrix and 100 μl plasma validation sample) was added to 4.80 ml of InISS and mixed for 30 min. An aliquot of each processed sample was then analyzed by ICP–MS together with QC samples and a multilevel calibration.

Intracellular antimony concentration was calculated by dividing the total estimated antimony content in the cell pellet by the total cell volume (number of PBMCs per pellet × 283 fL, where 283 fL is the average volume of a single PBMC [24]).

### Dilution

A 1:10 dilution of the samples was prepared by adding 0.5 ml of each digested sample to 4.5 ml of InISS. Samples were vortex mixed for approximately 10 s, tumbled for at least 30 min on rotary mixer and then transferred to a sample rack for analysis by ICP–MS.

## Results

### Assay specificity & calibration

**Figure 2:**
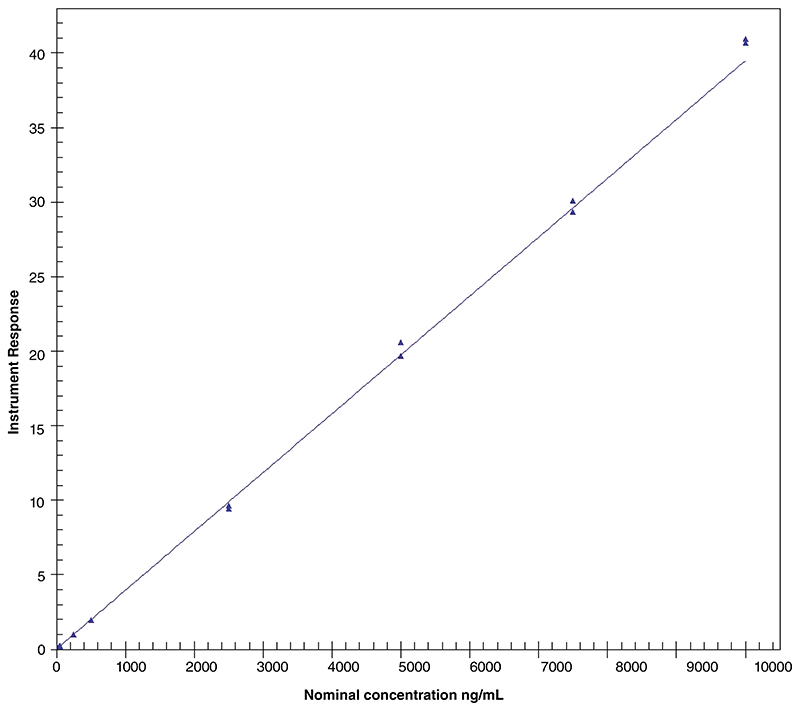
Representative calibration curve for antimony in human plasma. Calibration standards for antimony (25, 50, 250, 500, 2500, 5000, 7500 and 10000 ng/ml) were quantified using inductively coupled plasma mass spectrometry and calibration was fitted with a linear model based on weighting of 1/concentration^2^. This calibration was obtained from the analytical run 1 and processed with the software Watson LIMS version 7.5 SP1.

The spectrographic interference from antimony on the indium response was within acceptance criteria (≤5.00% of the mean peak area of indium matrix blank response in the run). Similarly, the spectrographic interference from indium on the antimony response was within acceptance criteria (≤25.0% of the mean peak area of the LLOQ of antimony response in the run). A linear model method using a weighting of 1/concentration^2^ was selected for calibration of antimony in human plasma and a representative calibration curve is presented in Figure 2.

The calibration curve parameters obtained throughout the validation study are listed in Table 3.

QC samples, prepared in plasma at antimony concentrations 50, 500, 7500 ng/ml were used to demonstrate the performance of the analytical assay. The relative bias of the back-calculated concentrations of antimony within the acceptance criteria ranged between -2.60 and 3.51% for 7 analytical runs ran over 132 days (Table 4).

QC data was obtained during the validation process. QC samples at low (50 ng/ml), medium (500 ng/ml) and high (750 ng/ml) concentration were evaluated by duplicate over analytical runs 2–6 (n = 10). The mean antimony concentration for the low concentration control was 50 ng/ml (SD = 3.89; %CV = 7.78; %Bias = 0.00); 494 ng/ml (SD = 9.91; %CV = 2.01; %Bias = -1.20) for the medium concentration control and 7520 ng/ml (SD = 189; %CV = 2.51; %Bias = 0.27) for the high concentration control sample. The impact of carry-over was assessed at the start and the end of every run containing a calibration curve. The mean percentage carry-over was 10.6 % and within the acceptance criteria (≤25.0% difference).

### Assay variability

Accuracy and within-run precision for the validation of the antimony assay are summarized in Table 5. The accuracy ranged from -1.20 to 1.20% of nominal values. At all concentration levels of antimony, the ranges of accuracy and precision were within established acceptance criteria.

Accuracy and within-run precision for the validation of the antimony assay in PBMC are summarized in Table 6. The accuracy ranged from -12.2 to 20.8% of nominal values. At all concentration levels of antimony, the ranges of accuracy and precision were within established acceptance criteria.

### Antimony stability

The stability of antimony in plasma was established following various storage conditions relevant to clinical sample handling. Antimony stability was evaluated at room temperature for 24 h using plasma samples spiked with 50 or 7500 ng/ml of antimony standards defined as low and high concentration quantification controls, respectively. Six replicates were analyzed for each concentration and condition. No significant changes in the plasma antimony concentrations were observed after this storage period. The mean concentration for the low concentration control was 50.4 ng/ml (SD = 4.28; %CV = 8.49; %Bias = 0.80) and 7550 ng/ml (SD = 123; %CV = 1.63; %Bias = 0.67) for the high concentration control.

Stability of antimony in processed human plasma samples after storage at room temperature for 120 h and 35 days at room temperature was also evaluated. Six replicates were analyzed for each concentration. Storage for 120 h resulted in a mean concentration for the low concentration control of 52.0 ng/ml (SD = 2.47; %CV = 4.75; %Bias = 4.00) and 7820 ng/ml (SD = 128; %CV = 1.64; %Bias = 4.27) for the high concentration control. Following storage of the processed samples for 35 days at room temperature resulted in a mean concentration for the low concentration control of 48.9 ng/ml (SD = 1.14; %CV = 2.33; %Bias = -2.20) and 7630 ng/ml (SD = 158; %CV = 2.07; %Bias = 1.73) for the high concentration control.

Stability of antimony in plasma was also established following storage at approximately -20°C and -80 ±10°C for 56 days. Six replicates were analyzed for each concentration and condition and the accuracy and precision for quantitation of antimony under these conditions was evaluated. Storage of plasma samples at -20°C for 56 days resulted in a mean concentration for the low concentration control of 48.7 ng/ml (SD = 1.66; %CV = 3.41; %Bias = -2.60) and 7450 ng/ml (SD = 80.7; %CV = 1.08; %Bias = -0.67) for the high concentration control. Storage at -80°C for the same period resulted in a mean concentration for the low concentration control of 48.2 ng/ml (SD = 2.30; %CV = 4.77; %Bias = -3.60) and 8150 ng/ml (SD = 315; %CV = 3.87; %Bias = 8.67) for the high concentration control.

Finally, stability of antimony in plasma was assessed following four freeze–thaw cycles at -20 and -80°C ±10°C utilizing plasma samples spiked with 50 or 7500 ng/ml of antimony standard. Six replicates were analyzed for each concentration and condition and the accuracy and precision for quantitation of antimony under these conditions was evaluated. Subsequent to four freeze–thaw cycles at -20°C the mean concentration for the low concentration control was 48.3 ng/ml (SD = 2.09; %CV = 4.33; %Bias = -3.40) and 7290 ng/ml (SD = 314; %CV = 4.31; %Bias = -2.80) for the high concentration control. At -80°C the mean concentration for the low concentration control was 46.3 ng/ml (SD = 1.18; %CV = 2.55; %Bias = -7.40) and 7530 ng/ml (SD = 107; %CV = 1.42; %Bias = 0.40).

### Influence of dilution on the quantitation of antimony in plasma

Plasma antimony levels can reach peak concentrations over 40 μg/ml following intramuscular administration of meglumine antimoniate [11], therefore plasma dilution is usually required for antimony quantification. The accuracy and precision results for quantitation of antimony following plasma sample dilution were evaluated over one analytical run and six replicates were measured. Plasma concentrations up to 100000 ng/ml were reliably analyzed when diluted 1:10 (v:v) into the calibration range of the assay. The mean quantified concentration of spiked plasma was 105000 ng/ml (SD = 2400; %CV = 2.29; %Bias = 5.00).

The accuracy and precision results for quantitation of antimony following extraction on diluted samples were also evaluated. Concentrations up to 100000 ng/ml were reliably analyzed when diluted 1:10 (v:v) into the calibration range of the assay. The mean concentration was 102000 (SD = 816; %CV = 0.80; %Bias = 2.00, n = 6).

### Antimony determination in clinical samples of patients undergoing antileishmanial treatment with meglumine antimoniate

**Figure 3:**
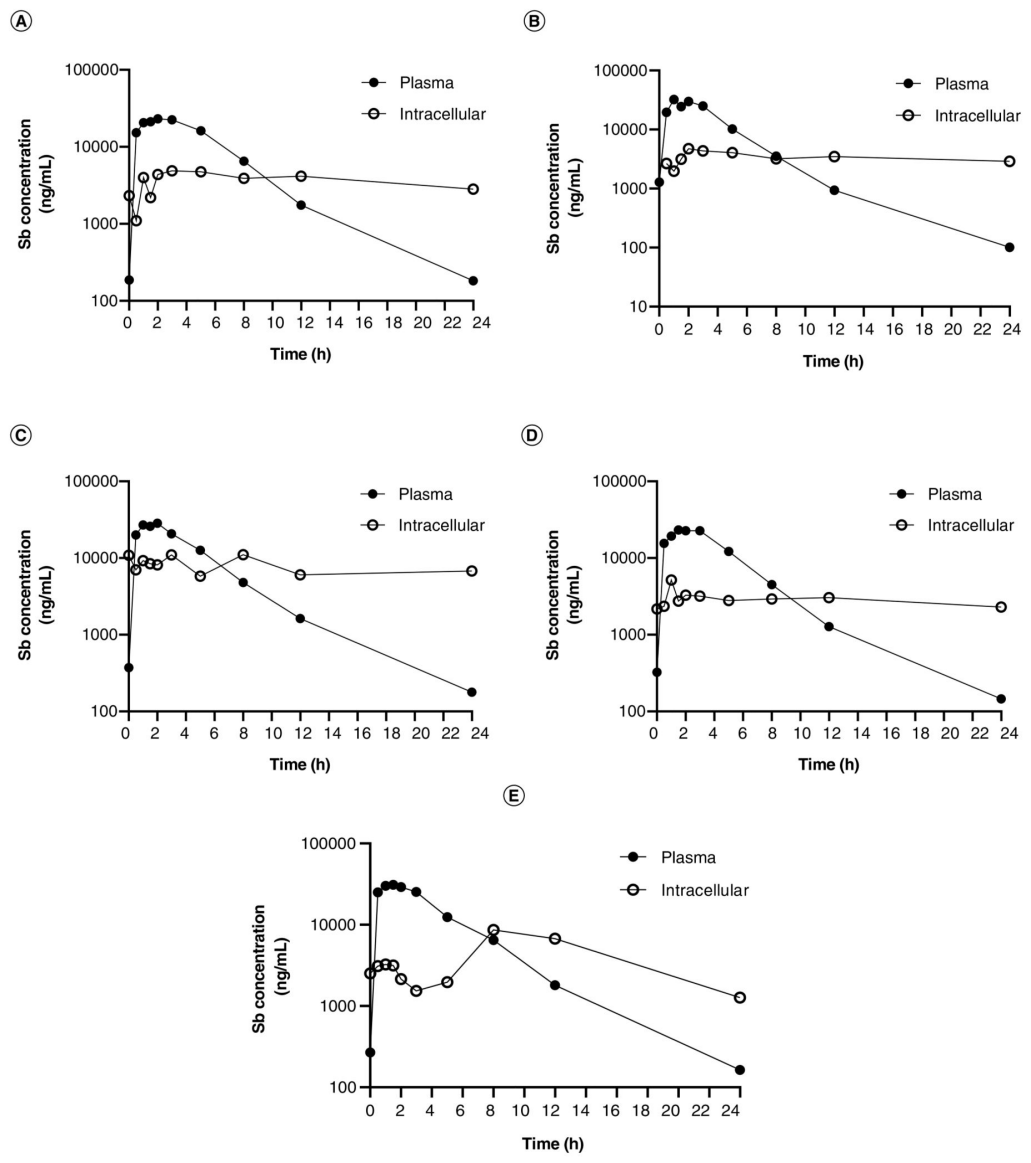
Antimony plasma and intracellular concentration–time curves. **(A–E)** Pharmacokinetic curves for antimony plasma and intracellular (peripheral blood mononuclear cell) levels. Samples were procured from five patients undergoing antileishmanial therapy with meglumine antimoniate. Samples were collected during the last day of treatment (day 20) and evaluated up to 24 h following drug administration.

The ICP–MS quantitative method validated in this study was applied to determine the plasma and intracellular levels of antimony in patients undergoing treatment for CL with meglumine antimoniate. Levels of antimony were measured at the end of the treatment (day 20) and evaluated up to 24 h following drug administration. Figure 3A–E presents the plasma and intracellular (PBMCs) concentration–time curves of five adult patients.

## Discussion

A wide range of analytical strategies have been used to quantify antimony in biological samples, and atomic absorption spectroscopy, using diverse atomization configurations, is the most employed technique [25,26]. Previously, our group standardized and applied an electrothermal atomic absorption spectrometry method to quantify antimony in plasma and urine samples after administration of intramuscular Glucantime [11]. However, no standardized methods for determination of intracellular concentrations of antimony were available to date, which limited the study of intracellular PKs of antimonial compounds at the site of drug action during antileishmanial chemotherapy.

We have developed and scientifically validated a reproducible and accurate ICP–MS method for the quantification of total antimony in human plasma and PBMCs in accordance with the European Bioanalysis Forum recommendations. Antimony levels in clinical samples were quantitatively determined by ICP–MS following digestion in 1% TMAH/1% EDTA, using indium as the internal standard. Accuracy and within-run precision were within acceptance limits. The maximum run size for this assay (assay robustness) was 240 injections per batch. Antimony was stable in human plasma at room temperature for at least 24 h, at approximately-20 or -80°C ±10°C for at least 56 days and following at least four freeze–thaw cycles. In addition, processed samples were stable for at least 35 days at room temperature. Dilution evaluation demonstrated that concentrations up to 100000 ng/ml of antimony could be reliably analyzed when diluted into the calibration range. These data show that our method has adequate specifications to reliably perform PK studies of total plasmatic and intracellular antimony. Accuracy and precision for PBMC was not as high as in plasma, but met the predefined scientific validation assay acceptance criteria (±25% at LLOQ). Table 7 presents a comparison of validated methods available for antimony determination in biological samples and the method we developed.

The main limitation of this method is that it does not allow Sb^III^ and Sb^V^ speciation. Antimony-based an-tileishmanial therapy is administrated as pentavalent antimony, which is rapidly absorbed reaching peak plasma concentration between 0.5 to 2 h. However bio-reduction to the trivalent form is required for the antileishmanial activity [9]. It has been proposed that this redox process can occur in the host cell phagolysosome where the parasites reside, or in the parasitic cytosol [31]. Sb^III^ induces an increase of intracellular Ca^2+^and finally apoptosis. Thus, antileishmanial activity is highly dependent upon intracellular mechanisms. Although this method cannot discriminate antimony species, this is the first validated method available for intracellular quantification of antimony.

We generated PK curves of antimony in PBMCs from patients undergoing meglumine antimoniate antileishmanial treatment, from samples obtained at the last day of treatment, and compared them to plasma concentration–time curves. Intracellular antimony concentration was over seven-times higher than plasma before the last dose was administered showing intracellular accumulation over the course of the antileishmanial treatment. As expected, a faster peak concentration was reached in plasma compared with PBMCs after the last dose was administered. Intracellular drug quantification in chemotherapy against intracellular pathogens is key to dissect the mechanisms of susceptibility and therapeutic response, since adequate drug levels at the effect site, the cells in which the pathogen survives and replicates, determine the pharmacological activity. Despite this, the relationships between plasma and intracellular drug accumulation remain unknown for leishmaniasis and PK studies rely on plasma drug concentrations assuming these act as surrogates of intracellular concentrations [32]. However, intracellular drug penetration can widely vary depending on host factors such as permeability, local metabolism and drug efflux/uptake transporters activity [33], which in turn, determine the level of exposure of intracellular pathogens to drugs. Additionally, multiple reports have shown a link between the host immune response and the antimonial therapy efficacy [34,35]. Therefore, liking information about intracellular drug concentrations, host immune signals and therapy outcome can provide a powerful framework to optimize therapeutic regimens for *Leishmania*, as well as for other intracellular pathogens.

## Conclusion

We have developed and validated an ICP–MS method in accordance with the European Bioanalysis Forum recommendations for the quantification of antimony in human plasma and PBMCs following digestion in 1% TMAH/1% EDTA and using indium as the internal standard. This method was successfully applied to determine PK curves of plasma and intracellular antimony concentrations in patients with CL undergoing treatment with meglumine antimoniate. We aimed to provide a reference method to allow detailed PK studies of intracellular antimony established during the course of antileishmanial chemotherapy.

## Future perspective

Treatment of CL relies heavily on few therapeutic options, and pentavalent antimonials remain as the first-line drugs, despite high toxicity, variable efficacy and increased resistance. Improvement of drug regimens has been hampered by limited understanding of detailed intracellular mechanisms of drug action, including PK events. Future applications of validated analytical methods that allow intracellular determination of antimony, including speciation, will contribute to characterize the relationship between antimony bioavailability, parasite clearance and immunomodulation, which are critical for understanding the determinants of therapeutic outcome and optimization of drug regimens.

## Figures and Tables

**Figure 1:**
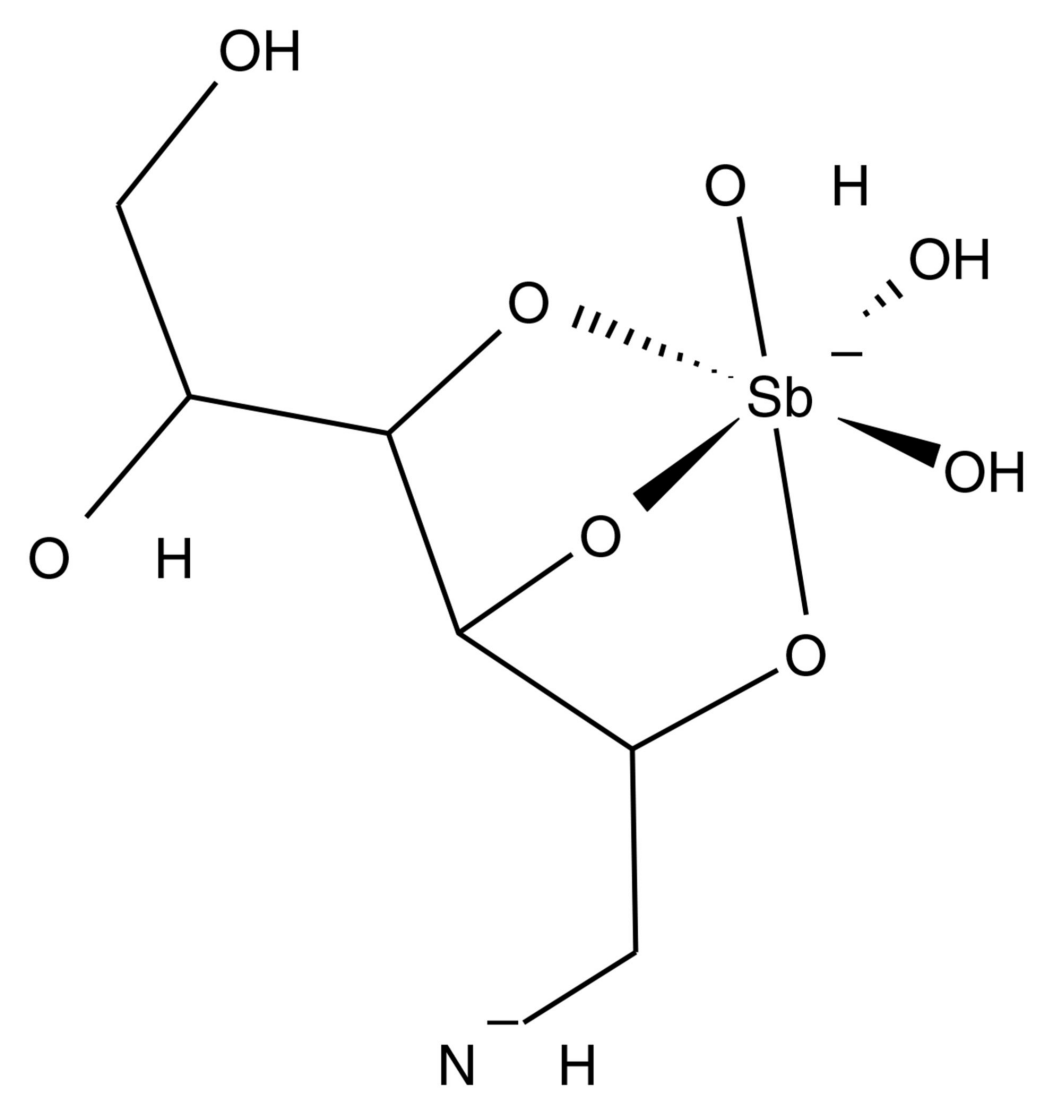
Proposed meglumine antimoniate chemical structure. Proposed structural formula for meglumine antimoniate (364 Da) in aqueous solution. Adapted with permission from [23].

**Table 1:** Clinical characteristics of cutaneous leishmaniasis patients.

Sociodemographic characteristics	n (%), n = 5
Age, mean (SD)	30.2 (12.61)
Sex, n (%):	
– Male	4 (80)
– Female	1 (20%)
Race, n (%):	
– Aboriginal	3 (60)
– Mestizo	2 (40)
Clinical outcome, n (%):	
– Cure	5 (100)
Patients reporting ADR, n (%):	
– At least one	5 (100)
Intensity of ADR, n (%):	
– Mild	4 (80)
– Moderate	1 (20)
Most common type of reaction, n (%):	
– Elevated amylases	3 (60)
– Decreased leukocyte count	3 (60)
– Headache	3 (60)
– Myalgia	3 (60)
Isolated species:	
– *Leishmania (Viannia) braziliensis*	3/5
– *Leishmania (Viannia) panamensis*	1/5
– Not available	1/5

ADR: Adverse drug reaction; SD: Standard deviation.

**Table 2:** Agilent 7700x operating parameters.

Parameter	Setting
**ICP–MS**	
RF power	1550 W
# of points per peak	3
Replicates	3
Integration time/mass	0.09 s (^115^In); 0.09 s (^121^Sb)
Total acquisition time	1.98 s
Nebulizer/carrier gas flow	1.05 l/min
Dilution mode	OFF
**Integrated sample introduction system (ISIS-DS)**	
Load time	8 s
Load speed	0.45 rps
Probe rinse time	90 s
Post rinse time	30 s
Post rinse speed	0.5 rps
Loop tubing ID	2.29 mm

ICP–MS: Inductively coupled plasma mass spectrometry; ISIS–DS: Integrated sample introduction system with discrete sampling; RF: Radio frequency.

**Table 3:** Calibration curve regression parameters for antimony.

Run day	Analytical run number	Slope	Intercept	R-squared	LLOQ	LOQ
1	1	0.0039481	0.0089183	0.9966	25	10000
1	2	0.0038862	0.0082196	0.9979	25	10000
40	3	0.0039172	0.018297	0.9969	25	10000
68	4	0.0032206	0.0037602	0.9981	25	10000
68	5	0.0031721	0.0065024	0.9981	25	10000
75	6	0.0035459	0.0058869	0.9964	25	10000
132	7	0.00386	0.022976	0.9953	25	10000
Mean		0.0036500	0.0106515	0.9970		
SD		0.0003376	0.0071493	0.0011		
%CV		9.25	67.12	0.11		

LLOQ: Lower limit of quantitation; LOQ: Limit of quantitation; SD: Standard deviation.

**Table 4:** Back-calculated calibration standards for antimony in human plasma.

Analytical run number	25.0 ng/ml	50.0 ng/ml	250 ng/ml	500 ng/ml	2500 ng/ml	5000 ng/ml	7500 ng/ml	10000 ng/ml
1	21.9	48.5	246	494	2390	4980	7420	10300
	28.0	52.2	250	489	2440	5210	7630	10400
2	23.8	50.2	238	501	2470	4990	7370	10600
	27.1	47.2	243	485	2480	5180	7780	10200
3	23.8	45.3	247	512	2420	5260	7430	10600
	27.6	49.0	245	514	2400	5180	7620	9580
4	26.0	49.7	252	504	2510	5350	7580	10700
	25.1	46.3	240	483	2460	5050	7120	9970
5	24.3	47.2	246	486	2500	5150	7460	10400
	26.6	48.7	261	512	2430	4880	7130	10600
6	23.4	49.5	249	463	2260	5350	7630	9410
	25.7	54.0	258	508	2500	5200	7680	^†^13400
7	23.6	50.2	254	503	2390	4920	7180	10900
	27.8	44.2	241	501	2490	4830	7670	10900
Mean	25.3	48.7	248	497	2439	5109	7479	10351
SD	1.92	2.61	6.69	14.3	66.2	169	215	462
%CV	7.59	5.36	2.70	2.88	2.71	3.31	2.87	4.46
%Bias	1.20	-2.60	-0.80	-0.60	-2.44	2.18	-0.28	3.51
n	14	14	14	14	14	14	14	13

Reason for exclusion.

† >20% of acceptance criteria, not in statistics.

SD: Standard deviation.

**Table 5:** Accuracy and precision of antimony in human plasma.

Analytical run number	25.0 ng/ml	50.0 ng/ml	500 ng/ml	7500 ng/ml
1	24.8	55.1	486	7420
	26.1	48.6	486	7600
	26.5	47.9	491	7570
	23.2	46	510	7630
	24.2	50.6	495	7610
	26.1	50.6	498	7680
Mean	25.2	49.8	494.0	7585
SD	1.30	3.13	9.05	88.71
%CV	5.16	6.29	1.83	1.17
%Bias	0.80	-0.40	-1.20	1.13
n	6	6	6	6

SD: Standard deviation.

**Table 6:** Accuracy and precision of antimony in human peripheral blood mononuclear cells.

Analytical run number	25.0 ng/ml	50.0 ng/ml	500 ng/ml	7500 ng/ml
7	27.6	43.8	437	6390
	31.3	42.3	454	6440
	34.3	45	446	7050
	28.4	44.2	420	7310
	29.3	44	454	7450
Mean	30.2	43.9	442	6928
SD	2.68	3.13	9.05	88.70
%CV	8.88	7.14	2.05	1.28
%Bias	20.7	-12.3	-11.6	-7.63
n	5	5	5	5

SD: Standard deviation.

**Table 7:** Validated methods for antimony quantification in human samples.

	Anodic stripping voltammetry	Inductively coupled plasma mass spectrometry and on-line ion chromatography	Graphite furnace atomic absorption	Electrothermal atomic absorption Spectroscopy	Hydride generation atomic fluorescence spectrometry	Inductively coupled plasma mass spectrometry
**Sample**	Heparinized blood	Heparinized blood, plasma, urine, hair	Urine	Plasma, urine	Hair	Plasma, PBMCs
**Amount of biological sample**	5 ml	Blood, plasma and urine: ND^†^ Hair: 0.5–2.0 cm length (measured from the scalp)	2g	ND	0.5 g	Plasma: 100 μl PBMCs: 2.27 ×10^6^–25.7 ×10^6^ cells
**Extraction or digestion methods**	ND^†^	Blood, plasma and urine: ND^†^ Hair: nitric acid and hydrogen peroxide	Chelation with thenoyl-trifluoro-acetone in supercritical carbon dioxide	Nickelous nitrate hexahydrate in nitric acid and Triton X-100	Nitiric acid, hydrochloric acid and water in closed system	1% TMAH/1% EDTA
**LLOQ**	76 ng/ml	0.16 ng/ml for Sb^V^ 0.15 ng/ml for Sb^III^	12.5 ng/ml	20 ng/ml	4.6 ng/g	25 ng/ml
**CV at lowest quantification**	1.3–3.4 %	0.9985 (R^2^) for Sb^V^ 0.9989 (R^2^) for Sb^III^	2.8% (RSD)	11.1%	2.8% (RSD)	Plasma: 5.16% PBMCs: 8.88%
**Quantification range of Sb concentrations**	76–29,500 ng/ml	0–100 ng/ml for both Sb^V^ and Sb^III^	2.5–200 ng/ml	0–200 ng/ml	ND^†^	25–10000 ng/ml
**Sb^V^ and Sb^III^ speciation**	No	Yes	No	No	No	No
**Reference**	Chulay JD *et al.* (1988) [27]	Miekeley N *etal.* (2002) [12]	Hui-Ming L (2013) [28]	Cruz A *et al.* (2007) [11], de Aguilar MG *et al.* (2018) [29]	Cardozo MC *et al.* (2016) [30]	Current study

LLOQ: Lower limit of quantification; ND: Not described; PBMC: Peripheral blood mononuclear cell; TMAH: Tetramethylammonium hydroxide.

## Data Availability

The data that support the findings of this study are presented within the manuscript.

## Summmary Points

- Pentavalent antimonials are widely used as treatment for cutaneous leishmaniasis, however the pharmacokinetics (PK) of these drugs at the site of action is unknown.
- PK studies rely on plasma drug concentrations assuming these act as surrogates of intracellular concentrations.
- We have developed and validated a reproducible and accurate inductively coupled plasma mass spectrometry method for the quantification of total antimony in human plasma and peripheral blood mononuclear cells (PBMCs) in accordance with the European Bioanalysis Forum recommendations.
- Antimony was stable under the following conditions: in plasma samples up to 24 h at room temperature, 56 days at approximately -20 or -80°C and after 4 freeze–thaw cycles. In processed samples up to 35 days at ambient temperature.
- Taking the lower limit of quantitation to be the lowest validation concentration with precision and accuracy ≤20%, the current assay was successfully validated from 25.0 to 10000 ng/ml for antimony in human plasma and PBMC.
- Our method was successfully applied to determine PK curves of antimony in plasma and PBMC from patients undergoing treatment for with meglumine antimoniate.
